# Spatio-temporal persistence of zooplankton communities in the Gulf of Alaska

**DOI:** 10.1371/journal.pone.0244960

**Published:** 2021-01-22

**Authors:** Brian A. Hoover, Marisol García-Reyes, Sonia D. Batten, Chelle L. Gentemann, William J. Sydeman

**Affiliations:** 1 Farallon Institute, Petaluma, California, United States of America; 2 CPR Survey, Marine Biological Association, Nanaimo, British Columbia, Canada; 3 Earth & Space Research, Seattle, Washington, United States of America; University of Maine, UNITED STATES

## Abstract

Spatial structuring of mid-trophic level forage communities in the Gulf of Alaska (GoA) is poorly understood, even though it has clear implications for the health of fisheries and marine wildlife populations. Here, we test the hypothesis that summertime (May-August) mesozooplankton communities are spatially-persistent across years of varying ocean conditions, including during the marine heatwave of 2014–2016. We use spatial ordinations and hierarchical clustering of Continuous Plankton Recorder (CPR) sampling over 17 years (2000–2016) to (1) characterize typical zooplankton communities in different regions of the GoA, and (2) investigate spatial structuring relative to variation in ocean temperatures and circulation. Five regional communities were identified, each representing distinct variation in the abundance of 18 primary zooplankton taxa: a distinct cluster of coastal taxa on the continental shelf north of Vancouver Island; a second cluster in the western GoA associated with strong currents and cold water east of Unimak Pass; a shelf break cluster rich in euphausiids found at both the eastern and western margins of the GoA; a broad offshore cluster of abundant pelagic zooplankton in the southern GoA gyre associated with stable temperature and current conditions; and a final offshore cluster exhibiting low zooplankton abundance concentrated along the northeastern arm of the subarctic gyre where ocean conditions are dominated by eddy activity. When comparing years of anomalous warm and cold sea surface temperatures, we observed change in the spatial structure in coastal communities, but little change (i.e., spatial persistence) in the northwestern GoA basin. Whereas previous studies have shown within-region variability in zooplankton communities in response to ocean climate, we highlight both consistency and change in regional communities, with interannual variability in shelf communities and persistence in community structure offshore. These results suggest greater variability in coastal food webs than in the central portion of the GoA, which may be important to energy exchange from lower to upper trophic levels in the mesoscale biomes of this ecosystem.

## Introduction

Long-term macro-scale ecological studies are rare [1] but are needed to investigate the impacts of global climate variability and change on terrestrial and aquatic ecosystems [2]. In marine ecosystems, zooplankton are thought to be one of the most responsive taxonomic groups to climate variability and change, and have been suggested as ecological “sentinels” [3–5]. In part, this is due to their small size, short lifespans and drifting life histories which should lead to responses to ocean climate change. Zooplankton form a major trophic link from primary producers to secondary and higher-level consumers, and thus are fundamental to marine food web dynamics and understanding fisheries and wildlife population fluctuations based on “bottom-up” consumer-resource functional relationships. Climate change impacts on wind patterns, ocean warming, and glacial run-off are expected to increasingly affect regional ocean conditions, including their water mass characteristics [6, 7]. These environmental changes may influence the spatial structure of zooplankton communities and thereby affect the nutritive value of zooplankton prey fields over large spatial scales [8]. It is expected that variability in currents and associated hydroclimatic variables are therefore likely to have strong effects on upper trophic level species and fisheries, operating directly or indirectly through spatial variation in zooplankton communities [9, 10].

While the effects of temperature and circulation on some zooplankton species’ distributions are relatively well known [11, 12], the effects of temperature variability on spatial structuring of zooplankton communities remains understudied. Within the Northeast Pacific Ocean (NEP), temperature effects on overall zooplankton community structure have been investigated within the contexts of size classes or regional and seasonal effects. For example, zooplankton sizes varied in response to temperature shifts between eastern and western north Pacific gyres [13], which may influence energy flow within local food webs. In a regional study on zooplankton community composition shifts, Eisner et al. [14] examined changes in large and small zooplankton assemblages within the eastern Bering Sea shelf, and found that larger copepods were more abundant under colder conditions in different regions of shelf habitat. In another study, four time-series of zooplankton data in the Gulf of Alaska (among other regions) showed major shifts in the phenology of seasonal abundance peaks across zooplankton taxa in relation to ocean surface temperature [15]; this result highlights the need to standardize seasonal effects when contrasting long-term community patterns. Within the context of the NEP, these examples show that while regional variability in zooplankton communities and long-term shifts in abundance patterns have been examined, long-term patterns in spatial variability of basin-scale zooplankton communities have not. Given the structuring effects of temperature and shelf bathymetry on zooplankton communities, there is a need to determine patterns of spatial persistence and change within the NEP.

In this paper we investigated the spatial variability in zooplankton communities by focusing on the linkages between ocean climate and multi-species abundances in the Gulf of Alaska (GoA), a region characterized by substantial interannual variation in circulation [16, 17] and ocean biogeochemistry [18]. As interannual variation in environmental conditions impact coastal, shelf, and off-shelf habitats differently, we expected that while zooplankton communities may shift in response to environmental variability, any shifts would consistently occur within spatially persistent habitat regions. Specifically, we hypothesized that the spatial distribution of zooplankton communities is consistent across years of varying ocean-climate conditions, including the period 2014–2016 when a large, anomalous marine heatwave (MHW) warmed the GoA pelagic environment 2–4 standard deviations above average [7]. To test this hypothesis, we analyzed 17 years of data on the distribution of zooplankton provided by the Pacific Continuous Plankton Recorder (CPR) program [19, 20, *https*:*//meetings*.*pices*.*int/projects/CPR#4*]. We identified key species that represent functional community groups, investigated spatial variation in summertime zooplankton assemblages across years, and related changes in spatial structure to ocean conditions, including variation in ocean temperatures and large-scale circulation. This approach represents the longest time-series analysis to date on large-scale zooplankton community patterns in the GoA, and the broad temporal and spatial data coverage allow us to examine the novel prediction that basin-scale community distributions will not respond to environmental change. Testing this hypothesis has significant implications for the wide variety of upper trophic level consumers in the GoA that directly or indirectly rely on zooplankton communities for sustenance, including fish [21, 22], seabirds [23, 24] and marine mammals [25, 26].

## Methods

### Data collection

**Fig 1:**
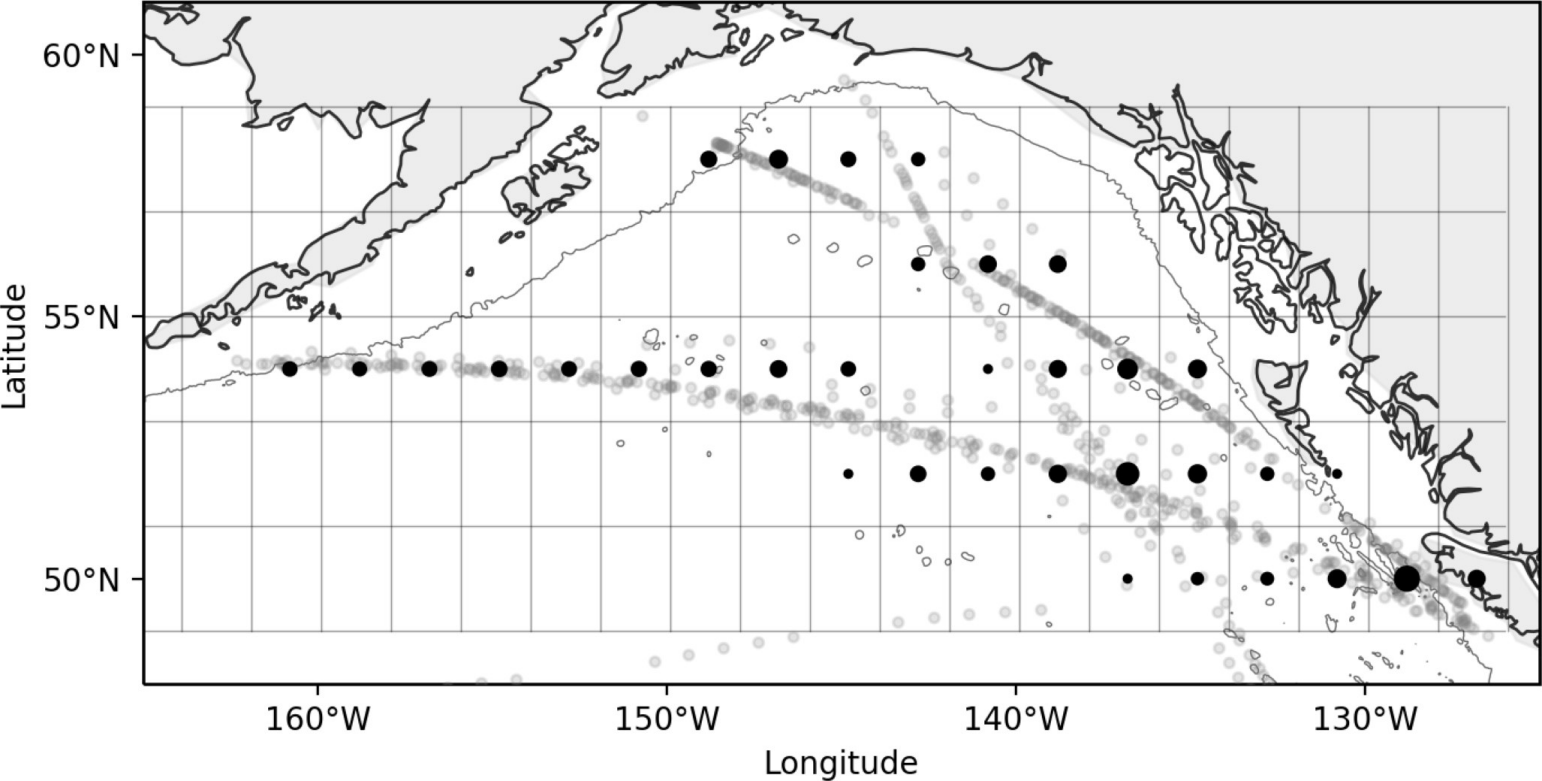
Map of the study area showing individual summertime CPR transects over 17 years (May 16-August 15, 2000–2016). Black circle within each grid cell depicts the center-point of the data (gray circles) which were averaged to produce abundance estimates for community analyses. Note that the center point is shown but sample locations may come from one part of the box only, especially for cells near to or including land. The size of each black circle scales to the sampling effort between grid cells. The coastal contour line depicts the 200 m isobath.

Continuous Plankton Recorder (CPR) sampling was conducted in the NEP in conjunction with commercial shipping operations, consisting of east to west transects extending from Juan de Fuca Strait to the south-central Bering Sea, and south-north transects extending from Juan de Fuca Strait to the northern GoA (Fig 1). CPR methodologies and data outputs have been previously reviewed [3, 19, 27–29]. Briefly, the CPR is towed behind a commercial ship and filters plankton from the upper layer of the ocean onto a length of filtering mesh. Given the typical vessel size and speed, although the CPR tows at a fixed depth of ~7 m, it effectively samples well-mixed surface waters to a depth of ~10 m [19]. The length of mesh is subsequently cut into discrete samples that each contain the plankton from 3 m^3^ of seawater collected over 18.5 km. CPR samples are nearly continuous, allowing for detailed analyses of zooplankton spatial organization. The aperture at the front of the CPR (1.2 cm sides) and the filtering mesh size within the device (270 μm) limit the upper and lower sizes of organisms that are quantitatively sampled. Furthermore, the formaldehyde preservative and turbulent nature of the sampling environment mean that fragile organisms are not well sampled. However, quantitative abundance and distribution information is obtained for robust mesozooplankton taxa (approximately 0.3 to 8 mm in length). Sampling typically begins in April each year and runs until about October. Here, we analyzed CPR data for samples collected during late spring and summer, i.e., mid-May through mid-August, 2000–2016. This period captures the peak in zooplankton abundance across the region as sampled by the CPR and is also least affected by weather-related sampling issues.

Collection of biological data in the US Exclusive Economic Zone to support fishery research is granted by the Magnuson—Stevens Fishery Conservation and Management Act. No other permitting for zooplankton collection or sample processing was required.

To standardize our analytical approach, we formatted data from each CPR sample within 2° latitude by 2° longitude grids of the GoA study domain. For each grid cell (hereafter called a “bin”) we calculated the mean abundance of each zooplankton taxon from all transects that passed through the bin, within and across years. To minimize potential sampling bias, bins containing fewer than 5 transects were excluded from analyses. To investigate geophysical covariates of zooplankton community composition across the domain, we integrated 3 environmental variables known to influence zooplankton abundance in this and other regions: i) water depth; ii) sea surface temperature (SST); and iii) current flow rates. To obtain coverage over data scarce regions, we used NASA satellite remotely-sensed SST and ocean currents over specific time periods. SST data were obtained from the NOAA Optimal Interpolation SST, a combined and corrected interpolation of data from different platforms (satellites, ships, buoys) with a daily resolution of 1/4° [30]. Ocean surface current data are from the Ocean Surface Currents Analysis Real-time (OSCAR, version 1) data set [31, 32]. OSCAR currents, representing the geostrophic and ageostrophic flow at 15 m depth, were calculated from satellite sea surface height gradients, ocean vector winds, and SST data every 5-days on a 1/3° global grid. Bathymetric data was obtained from ETOPO1 dataset, with a 1 arc-minute resolution (doi: 10.7289/V5C8276M).

Depth, SST and ocean currents were calculated by averaging data within each 2°x2° bin, from May 16—August 15, from 2000 to 2016 to match the temporal window of CPR data used in this study. To assess the effects of spatial and temporal variability in SST and ocean currents, we used the mean and standard deviation of each metric within each bin. We compare these values with the location of clusters identifying zooplankton community structure. To compare unusually warm and cold environmental years, annual anomaly values were calculated by averaging daily values per year (May 16—August 15) for all bins, and then removing the mean value of all years. Using this approach, we selected the five warmest and five coldest years for zooplankton community comparison (warm: 2004, 2005, 2014, 2015 and 2016; cold: 2007, 2008, 2010, 2011, 2012).

### Analysis of zooplankton communities and environmental covariates

Following standard CPR taxonomic protocols, zooplankton specimens are identified to the highest practical taxonomic resolution (Richardson et al., 2006); using this approach 119 zooplankton taxa were identified in our study samples. These taxa were subsequently collated into approximately 60 functional taxa groupings. Note that for small species all life history stages that are sampled are combined (although younger stages may not be retained by the mesh) and for larger species (e.g. copepods > 2 mm in length, euphausiids, chaetognaths) two groupings of juveniles and sub-adult/adult stages are typically made. Rare taxa, defined as those present in less than 15% of all bins, were excluded from analyses, yielding a total of 18 taxa that were used for community analyses in this paper. As the relative abundance of each taxon varied widely, we summarized the mean abundance of each taxon within each bin, and normalized these values to mean unit variance by subtracting the mean from each value and dividing by the standard deviation.

To establish a baseline community climatology over the entire 17-year study period, we used the normalized taxa abundance values associated with each bin to characterize community structure using Principle Component Analysis followed by Hierarchical Clustering of Principle Components (HCPC) [33]. These ordination and clustering techniques were applied to reduce the noise in these multivariate zooplankton data, and discern spatial patterns in community structure. We used broken stick models and Bayesian sensitivity analyses to first determine the appropriate number of principle components to retain for each analysis [34]. As our approach focuses on the most common zooplankton taxa and thus is not strongly skewed by zero values, we then applied hierarchical clustering on the selected components using Euclidean distance and Ward’s agglomeration criterion [35]. We assessed the inertia gain among varying numbers of clusters to verify the appropriate number of final clusters chosen, and used the explained variance in the clustering assignments to visualize the spatial patterns in 2° by 2° bins in a two-dimensional factor map. We determined the trends in the taxa that were most significantly associated with each spatial cluster, and mapped the final cluster designations onto a study map of the system.

### Analysis of environmental covariates of spatial variation in zooplankton communities

To assess the effects of upper ocean temperature on community structure, we examined spatial variation in community composition between anomalously cold and warm environmental periods, which we defined as years that varied more than +/- 0.5°C from baseline temperature climatology. We averaged the taxa across bins within cold and warm years, respectively, and then tested whether an overall community difference existed between these years using the permutational multivariate analysis of variance using distance matrices test (ADONIS) available in the R package ‘Vegan’, using average water depth (Depth) of each bin as an additional covariate. We investigated specific drivers of overall community differences using Indicator Species Analysis available from the R package ‘indicspecies’, and characterized community structure patterns in warm and cold years by applying a second HCPC analysis to determine whether community clusters persisted or changed between years of contrasting anomalous temperatures. For this second PCA, we pooled the taxa associated with cold and warm years together using a qualitative Temperature term, in addition to the quantitative factors depth, latitude, and longitude. Following model inspection, we kept the first ten Principle Components for hierarchical clustering, and otherwise followed the same protocol as the first analysis. Spatial ordination, clustering analyses and mapping were conducted in R Statistical Software, using the packages FactoMiner, FactoExtra, leaflet, and ggmap. Oceanographic climatologies were processed and mapped in Python.

## Results

**Fig 2:**
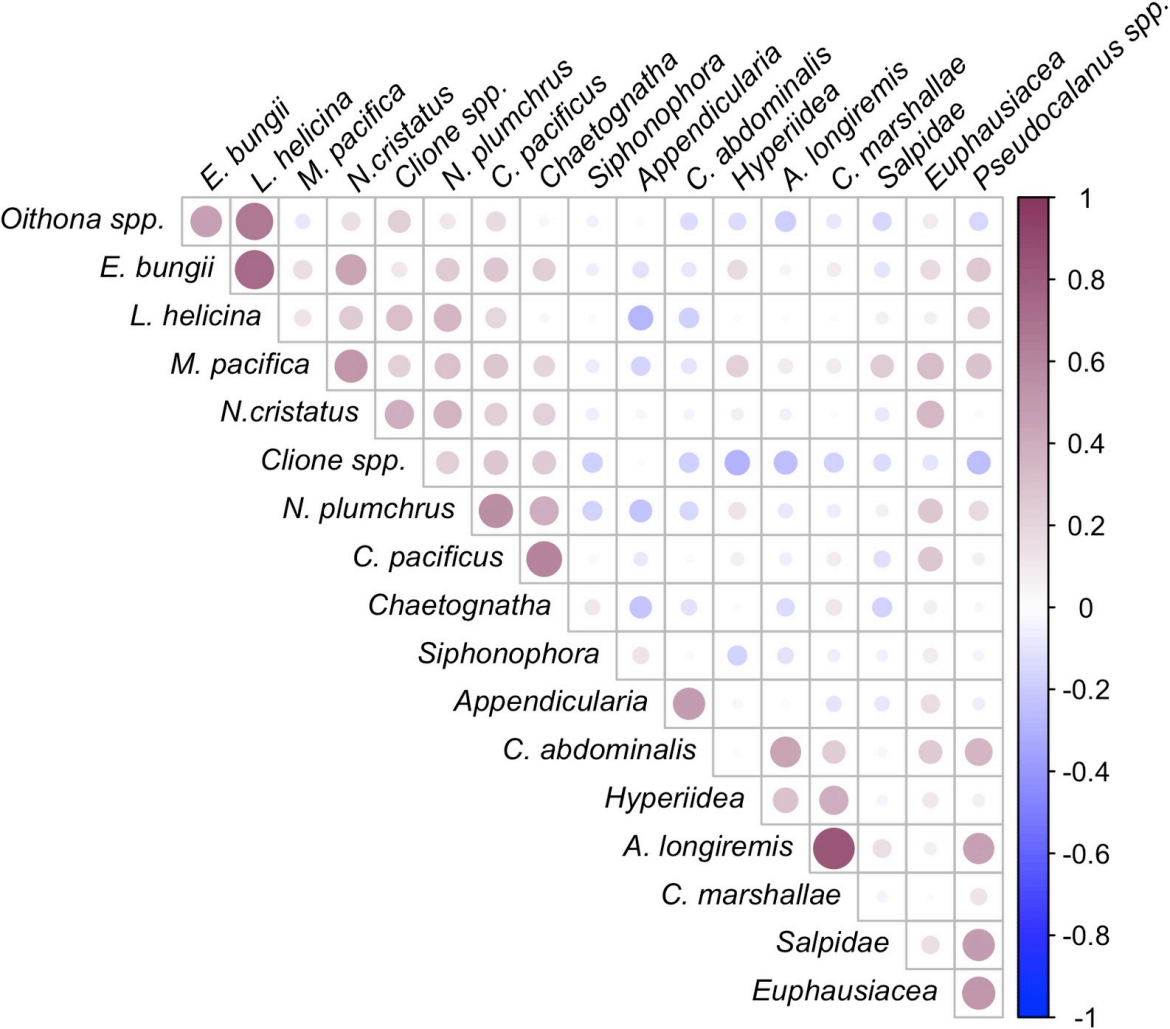
Pearson correlation coefficient between mean abundance of 20 zooplankton taxa used in this study. The size and color of circles are proportional to the strength of each correlation coefficient, with red representing positive correlations and blue negative correlations. (*p* < 0.01).

Taxonomic data from 2000–2016 May-August CPR transects were binned into 34 2° by 2° spatial bins throughout the Gulf of Alaska (Fig 1). The frequency of occurrence and taxonomic traits of the taxa selected for analysis are presented in Table 1. There were a few clear indications of covariance among the 18 selected taxa: strong positive correlations were found between *A*. *longiremis* and *C*. *marshallae* (0.83), *L*. *helicina* and *E*. *bungii* (0.73), *L*. *helicina* and *Oithona* spp. (0.66), and *C*. *pacificus* and chaetognaths (0.60) (Fig 2). No other correlations exhibited correlation coefficients greater than 0.6.

**Table 1:** The 20 dominant zooplankton taxa recorded in CPR transects, summarized by functional traits and frequency of abundance across 34 spatial bins and 17 years of data collection.

Taxa	% Spatial coverage	% Temporal coverage	Functional traits
			**Large off-shelf subarctic copepods**
*N*. *plumchrus/flem*. *(C3-C5)*	100	94	Primarily surface grazers
*E*. *bungii (C2-C6)*	88	94	Deep; detritus and particulate omnivores
*N*. *cristatus(C1-C6)*	97	100	Deep; detritus and particulate omnivores
			**Large on-shelf subarctic copepods**
*C*. *marshallae (C5-C6)*	79	100	Depth varies; grazers and omnivores
			**Mid-sized widespread copepods**
*M*. *pacifica (C5-C6)*	85	100	Strong diel migrators;
*C*. *pacificus (C5-C6)*	100	100	More abundant in warm years
			**Mid-sized on-shelf copepods**
*A*. *longiremis*	74	100	Subarctic, omnivorous
*C*. *abdominalis*	18	47	Subarctic, omnivorous
			**Small widespread copepods**
*Pseudocalanus* spp.	88	100	Wide temp. range
*Oithona* spp.	97	100	Wide temp. range
			**Pteropods**
*Clione* spp.	91	94	Feeds exclusively on *L*. *helicina*
*L*. *helicina*	100	100	Omnivorous
			**Gelatinous zooplankton, wide diversity of body types and life-histories.**
*Salpidae*	18	29	Solitary or colonial mucus filter-feeders
*Appendicularia*	91	100	Solitary filter-feeders
*Siphonophores*	18	25	Colonial carnivores
*Euphausiacea (adults)*	10	100	Krill; large diel migrators that may avoid CPR
*Hyperiidae*	100	100	Widespread amphipod crustaceans
*Chaetognaths (adults)*	100	100	Arrow worms, predators of small copepods

^a^If not otherwise indicated then all life history stages are included, but younger stages may not be captured by the CPR.

Six principle components were retained for cluster analysis (S1 Fig). The first six primary principle components explained 63.7% of the variation in overall zooplankton community structure (SI). PC1, which explained 19.8% of the variation in the community data, was strongly associated with longitude and depth, and corresponded to zooplankton of the coastal margins of the GoA. PC2 (15.9%) was most strongly associated with depth and latitude, and thus described the latitudinal gradient on coastal communities. The remaining components did not align with longitude, latitude or depth in obvious patterns, and were more difficult to interpret.

### Baseline zooplankton communities

**Fig 3:**
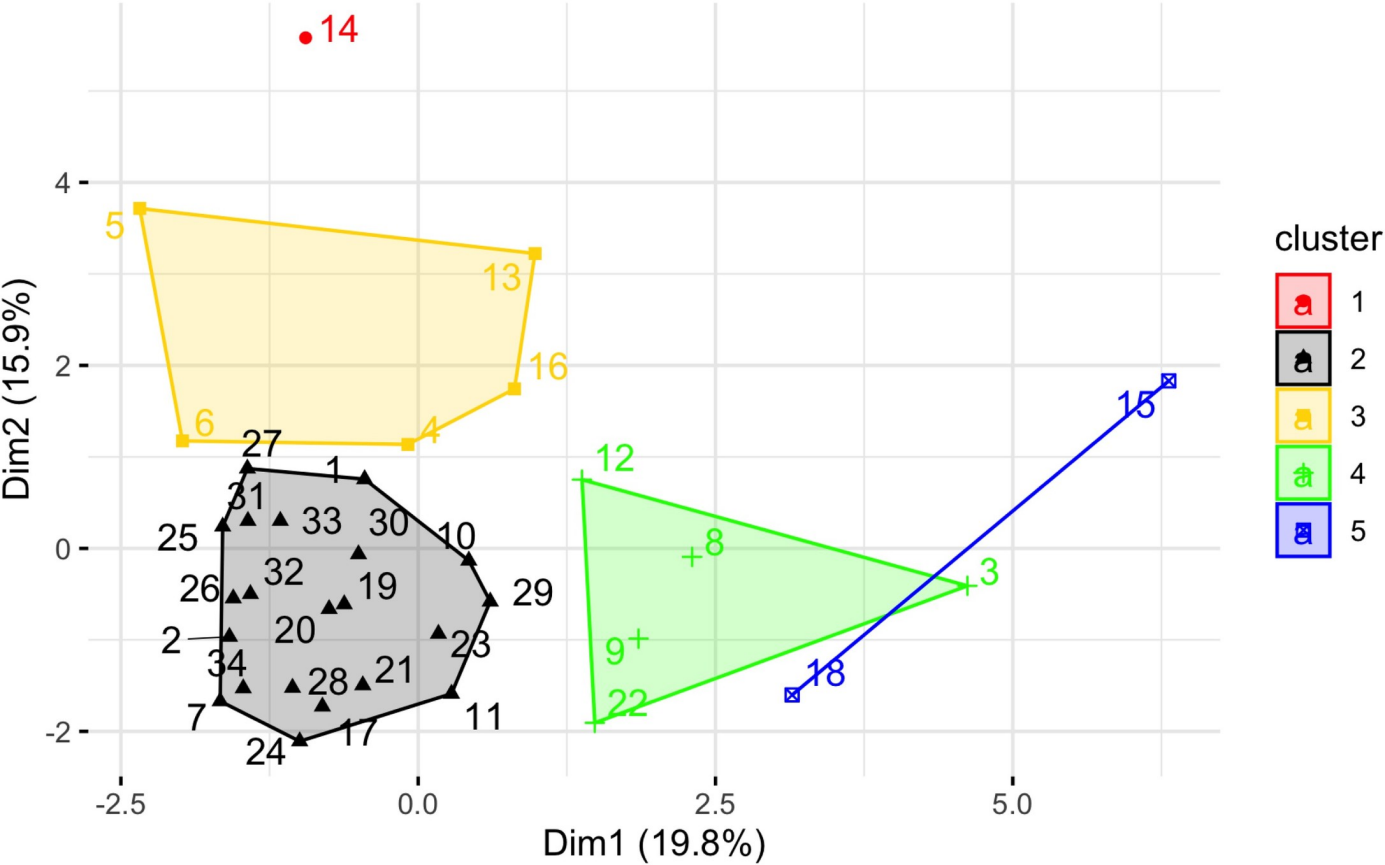
Clustering output from hierarchical clustering of principle components (HCPC) procedure. Two-dimensional (Dim1 and Dim2) factor map depicting the alignment of spatial bins into five distinct clusters along Principle Component axes 1 and 2, based on hierarchical clustering of the first six principle components.

**Fig 4:**
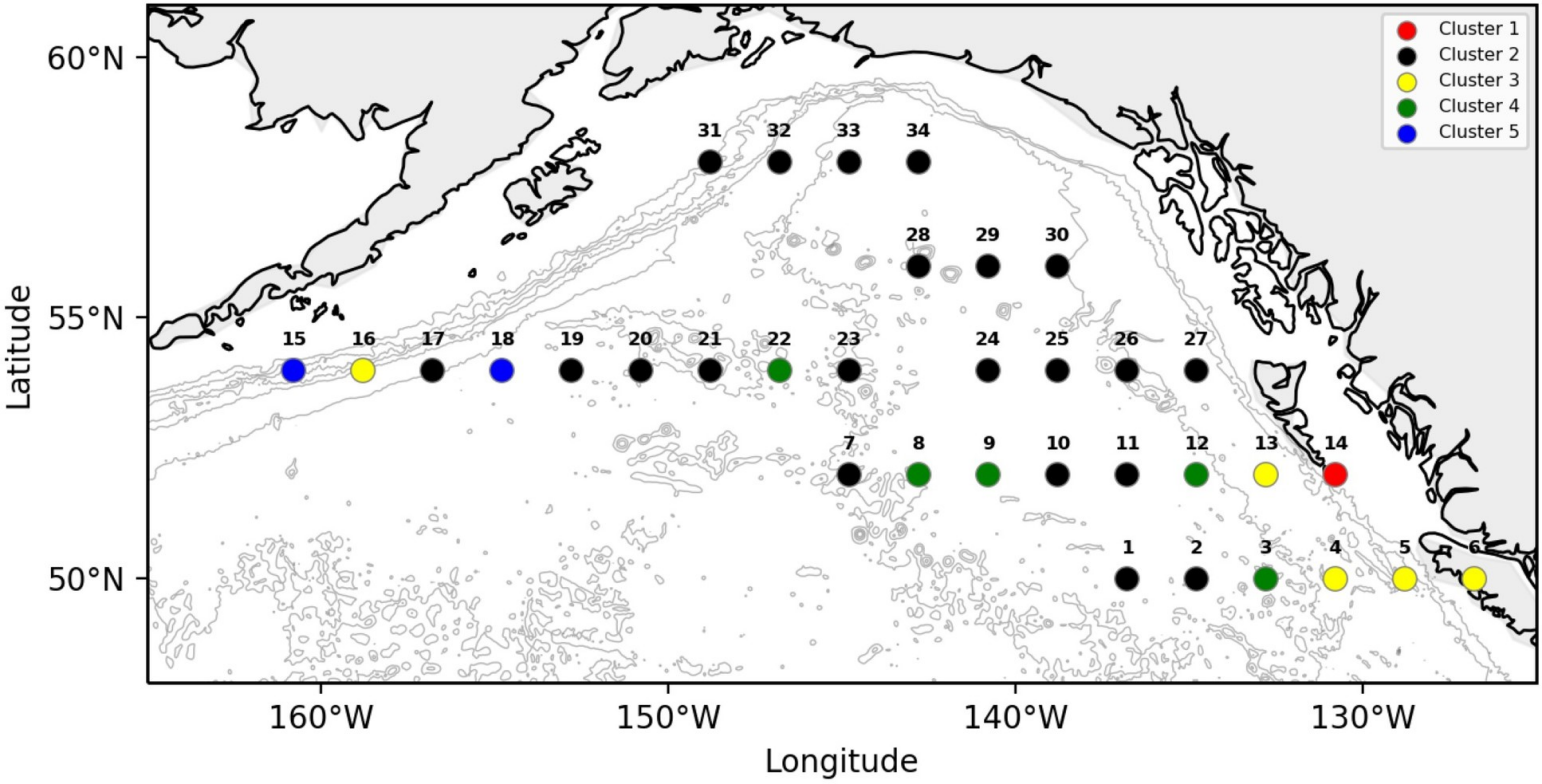
Spatial organization of five distinct clusters across the study region. The centerpoint of each bin is color-coded by cluster ID and mapped onto bathymetric contours depicting on-shelf and off-shelf regions of the GoA basin. Data points are numbered from East-West and South-North.

We used the loadings from the six principle components to cluster similarities among spatial bins and identify taxa significantly associated with each cluster (Table 2; Fig 3). Hierarchical clustering identified five discrete clusters, three of which were generally associated with coastal regions (Fig 4: Clusters 1, 3 and 5). Among these coastal clusters, Cluster 3 was represented on both coastal margins of the GoA, and was characterized by increased abundance of euphausiids, salps, and the copepod taxa *Pseudocalanus* spp. and *C*. *abdominalis*. In contrast, Clusters 1 and 5 occurred within specific geographic regions. Cluster 1 was found on the shelf north of Vancouver island, and was characterized by an increase in the neritic copepods *C*. *marshallae* and *A*. *longiremis*. Cluster 5 was represented by two bins east of Unimak Pass along the western GoA shelf, and was defined by an increased abundance of *L*. *helicina*, *Oithona spp*., *and E*. *bungii*. Clusters 2 and 4 represented offshore regions in the deep central GoA gyre. Cluster 4 was distributed within the southern basin, and was characterized by increased abundances of chaetognaths, *Clione* spp. pteropods, and the copepods *C*. *pacificus*, *N*. *cristatus*, *M*. *pacifica*, and *N*. *plumchrus/flemingiri* (hereafter shortened to *N*. *plumchrus*). Cluster 2 was distributed throughout the north-central GoA, where it was defined by a significantly decreased mean abundance within several taxa, including euphausids, chaetognaths, and the copepods *Pseudocalanus* spp., *N*. *cristatus*, *M*. *pacifica*, *C*. *pacificus*.

**Table 2:** Functional groups significantly associated with baseline spatial clusters.

Cluster	Taxa	V test	Mean (in cluster)	Mean (overall)	St. Dev. (overall)	p-value
1	*C*. *marshallae (C5-C6)*	5.65	77.4	3.49	13.08	*<0*.*0001*
	*A*. *longiremis*	4.60	240	28.50	45.9	*<0*.*0001*
2	*Euphausiids*	-3.58	3.41	6.29	5.87	*<0*.*001*
	*Pseudocalanus spp*.	-3.14	16.16	25.47	21.64	*0*.*002*
	*C*. *pacificus (C5-C6)*	-3.02	2.13	3.14	2.43	*0*.*003*
	*M*. *pacifica (C5-C6)*	-2.72	1.08	2.28	3.22	*0*.*007*
	*N*. *cristatus (C1-C6)*	-2.18	5.23	7.69	11.90	*0*.*03*
	*Chaetognaths*	-2.12	5.34	7.70	8.09	*0*.*03*
3	*Euphasusiids*	3.60	15.15	1.27	5.87	*<0*.*0004*
	*Pseudocalanus spp*.	3.55	57.67	25.47	21.64	*<0*.*0004*
	*Salps*	3.27	0.28	0.05	0.12	*0*.*03*
	*C*. *abdominalis*	2.48	8.72	1.77	6.69	*0*.*01*
4	*Chaetognaths*	3.70	20.26	7.70	8.09	*<0*.*0003*
	*C*. *pacificus (C5-C6)*	3.03	6.22	3.14	2.43	*0*.*003*
	*Clione* spp.	2.99	1.09	0.55	0.43	*0*.*003*
	*N*. *cristatus (C1-C6)*	2.72	22.36	8.79	11.90	*0*.*007*
	*M*. *pacifica (C5-C6)*	2.50	5.66	2.23	3.23	*0*.*01*
	*N*. *plumchrus (C3-C5)*	2.33	82.66	51.46	31.87	*0*.*02*
5	*L*. *helicina*	4.91	90.2	20.44	20.38	*<0*.*0001*
	*Oithona spp*.	4.19	158.75	40.76	40.40	*<0*.*0001*
	*E*. *bungii (C2-C6)*	4.15	13.3	1.53	4.07	*<0*.*0001*

^a^ The means and standard deviations of each taxon within its cluster is shown, along with its positive (+) or negative (-) relationship compared to its background abundance in all other clusters.

### Environmental climatology of zooplankton communities

**Fig 5:**
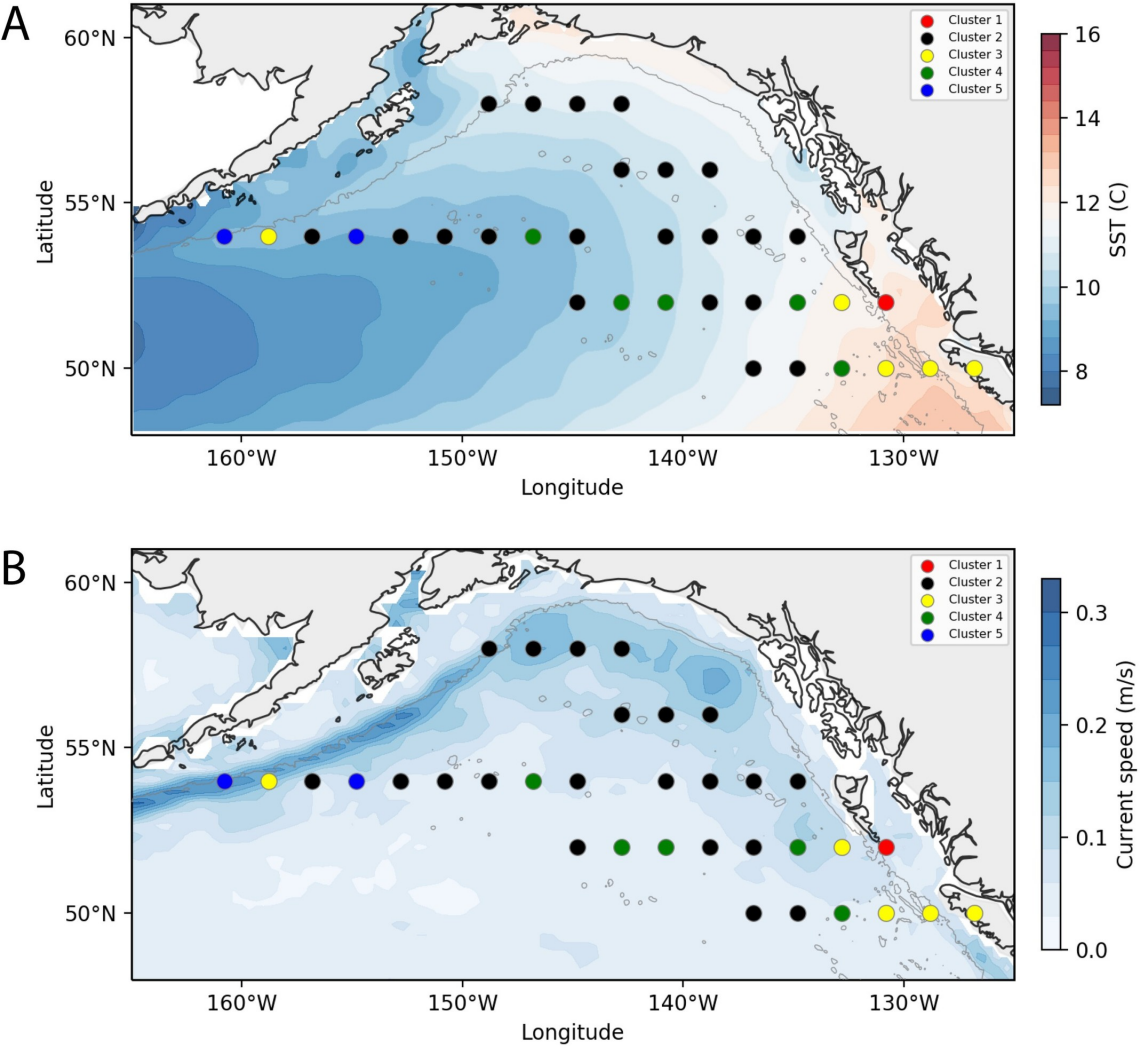
Climatology maps (May 16-August 15, 2004–2016). Spatial maps depicting (A) SST, (B) mean current speed. Dots indicate previously defined zooplankton community clusters (identified by color). Current speeds averaged in both meridional and zonal axes.

**Fig 6:**
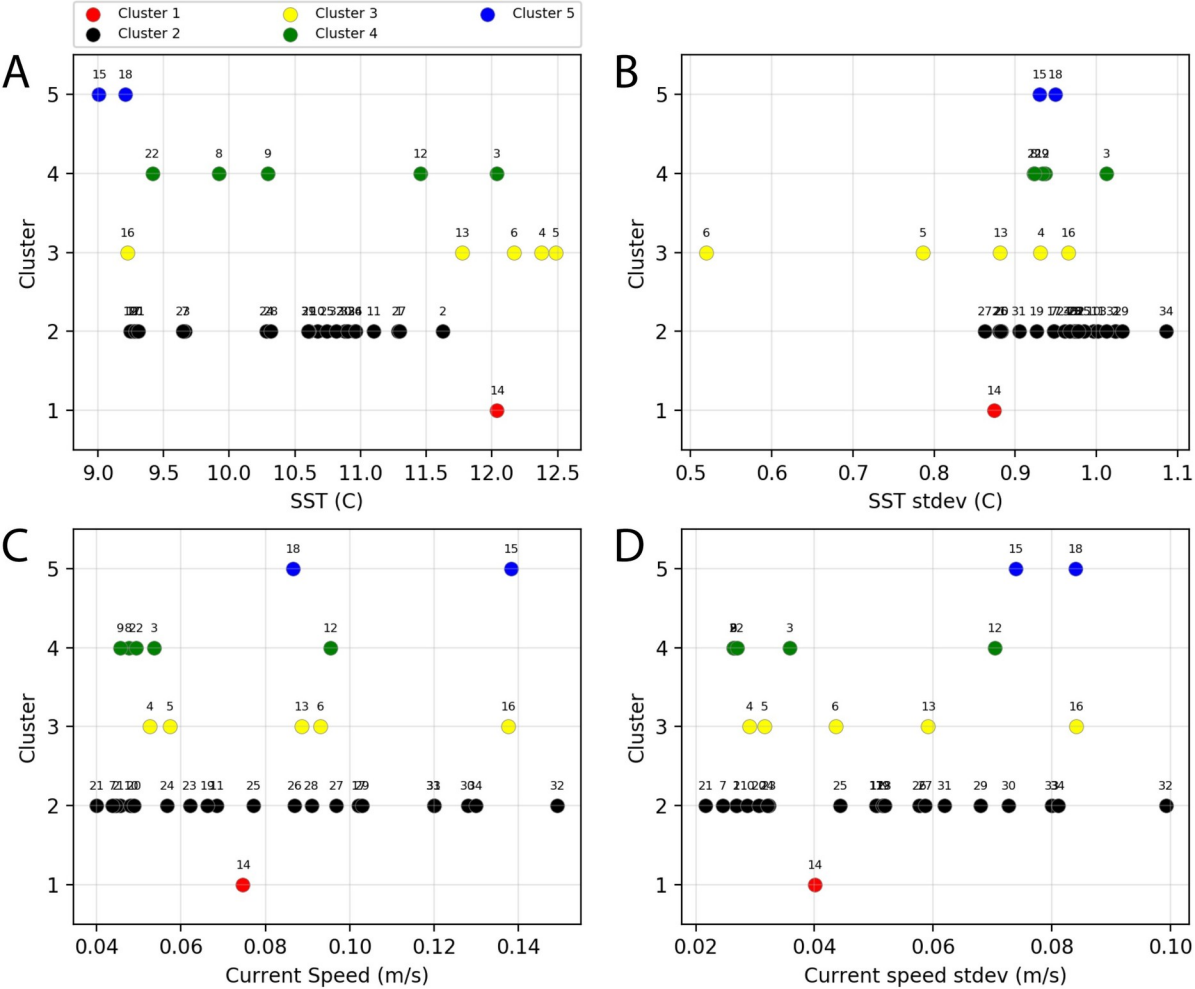
Relationship between community clusters and environmental variables. Distribution of (A) mean SST; (B) mean SST standard deviation; (C) mean current speed; (D) mean current speed standard deviation. All climatological means were produced for the period May 16-August 15, 2000–2016. The numbers near each data point refer to the Bin ID, as represented in Fig 4.

Temperature varied from the eastern to western GoA, with the coolest temperatures in the western GoA and the warmest in the eastern shelf. This temperature gradient was thus confounded with longitudinal geographic effects indicating that temperature was not clearly associated with patterns of overall community clustering (Fig 5A). Ocean currents show distinctive increases in speed within the Alaska current in the western GoA, whereas the central GoA gyre exhibited consistent slow speeds and low spatial variability (Fig 5B). Within each cluster, the distribution of data in SST, SST variability, current speed, and current variability was plotted (Fig 6), and summarized conceptually in Table 3. While most clusters did not show strong patterns associated with these variables, Cluster 5 encompassed the two bins with the lowest mean temperatures, whereas the nearshore Clusters 1 and 3 were more strongly associated with warm temperatures. Within the two central gyre clusters, Cluster 4 was generally associated with low current speeds and low current speed variance, whereas Cluster 2 varied widely in SST and current speeds.

**Table 3:** Description of geophysical characteristics associated with each identified zooplankton community cluster.

Geography/ Bathymetry	Cluster	SST(in summer)	Ocean Currents
Off-shelf, widely distributed throughout central GOA, deep waters	2	No pattern	No pattern
Off-shelf, southern GOA, deep waters	4	No pattern	Weak current speeds ($\bar{x}$ = 0.59 m/s)_and low variability associated with the interior of the Alaskan Gyre and the North Pacific Current
Off Haida Gwaii Island, shelf break (shallow)	1	Warm temperature (> 12°C) associated with the northeasterly North Pacific Current	Low-mid current speed (< 0.08 m/s), low variability, associated with the slow North Pacific Current
Both GoA coastlines, all depths, but near the coast	3	No pattern	No pattern
Western GoA near Unimak Pass, shelf break (shallow)	5	Cool temperatures (< 9.25°C) associated with the southwest bound Alaska Stream	Strong current speed (>0.14 m/s) and variability at Alaska Stream site near Unimak Pass (Bin 15),weaker current at offshore location (Bin 18).

### Interannual temperature variation and community structure

**Fig 7:**
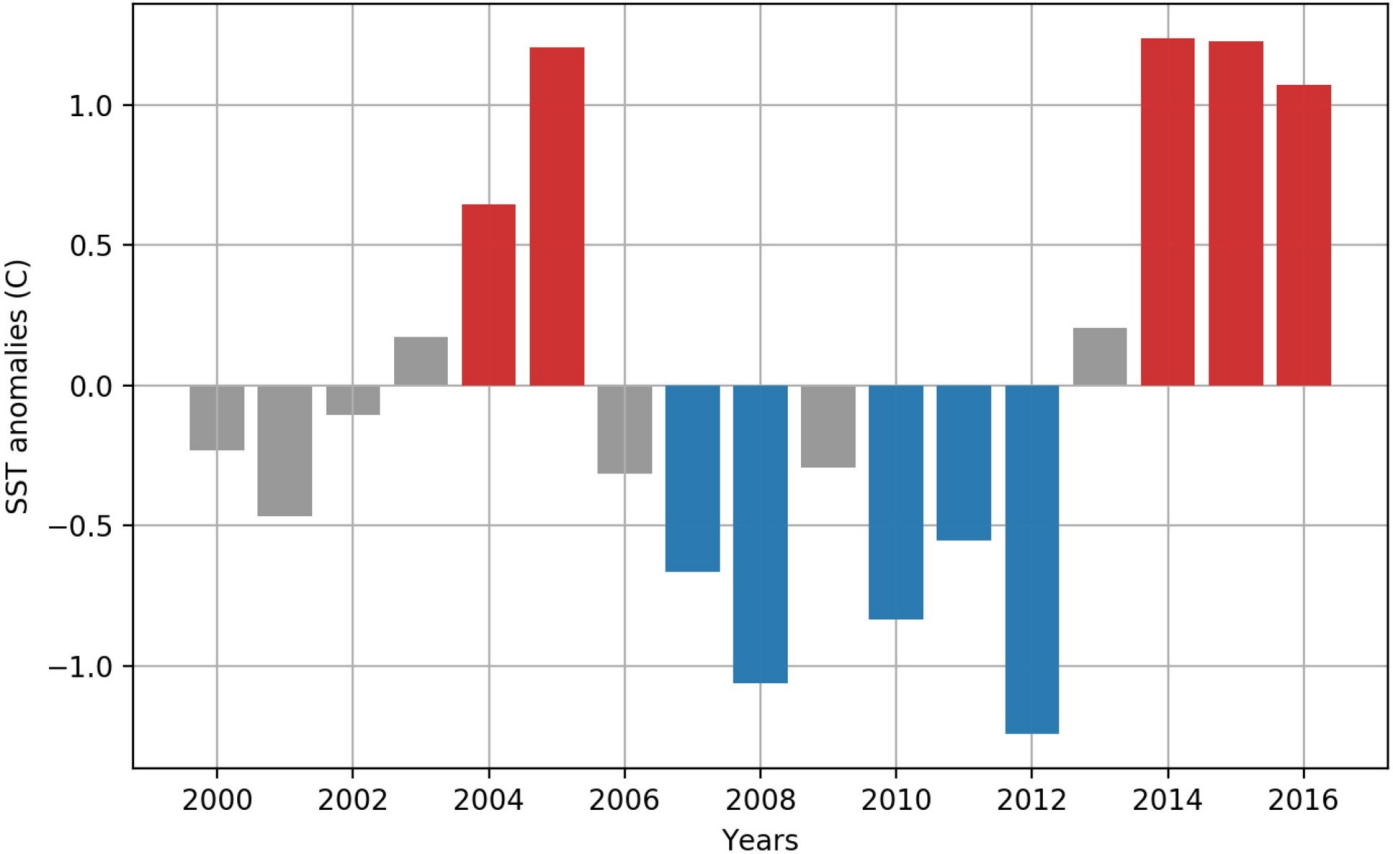
Selection of cold and warm years used for comparative community analysis. The five warmest and coldest years during which the SST anomaly deviated from the climatological (May 16—August 15) mean by 0.5°C were used to identify anomalous years (warm years: 2004, 2005, 2014, 2015, 2016; cold years: 2007, 2008, 2010, 2011, 2012). Years which do not identify as anomalous cold or warm years are shaded in gray.

**Fig 8:**
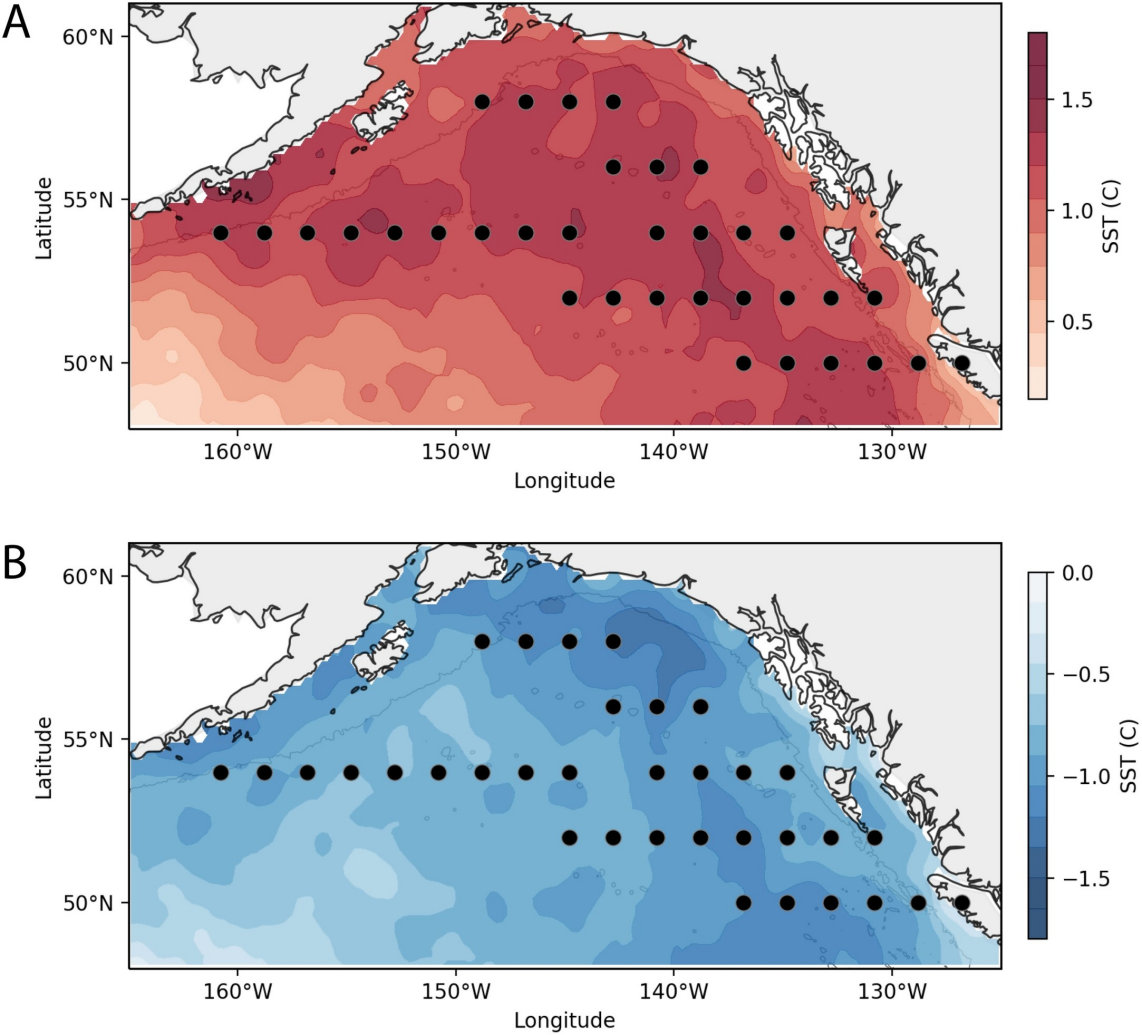
Temperature anomalies during warm and cold years. Study maps depicting SST patterns between (A) warm and (B) cold years, with center-points of sampled grids (black circles).

We identified the five coldest and five warmest years in which the mean SST deviated the most from the climatological mean, and extracted these anomalous warm (2004, 2005, 20014, 2015, 2016) and cold (2007, 2008, 2010, 2011, 2012) years for subsequent analysis (Fig 7). Temperature gradients within each subset of years varied strongly by region (Fig 8A and 8B). During cold years, the coldest regions were in the eastern GoA, whereas during warm years the warmest regions were broadly distributed within the central gyre.

There was no significant effect of temperature on overall zooplankton community structure when assessed across the entire study region (S1 Table: Permutational Multivariate Analysis of Variance; *p = 0*.*35*), though there was a strong structuring effect of depth (F = 3.63, *p = 0*.*001*) and a significant interaction between depth and temperature (F = 2.96, *p = 0*.*004*). Indicator Species Analysis did not identify any taxa that contributed significantly to whole zooplankton community differences between cold and warm years.

**Fig 9:**
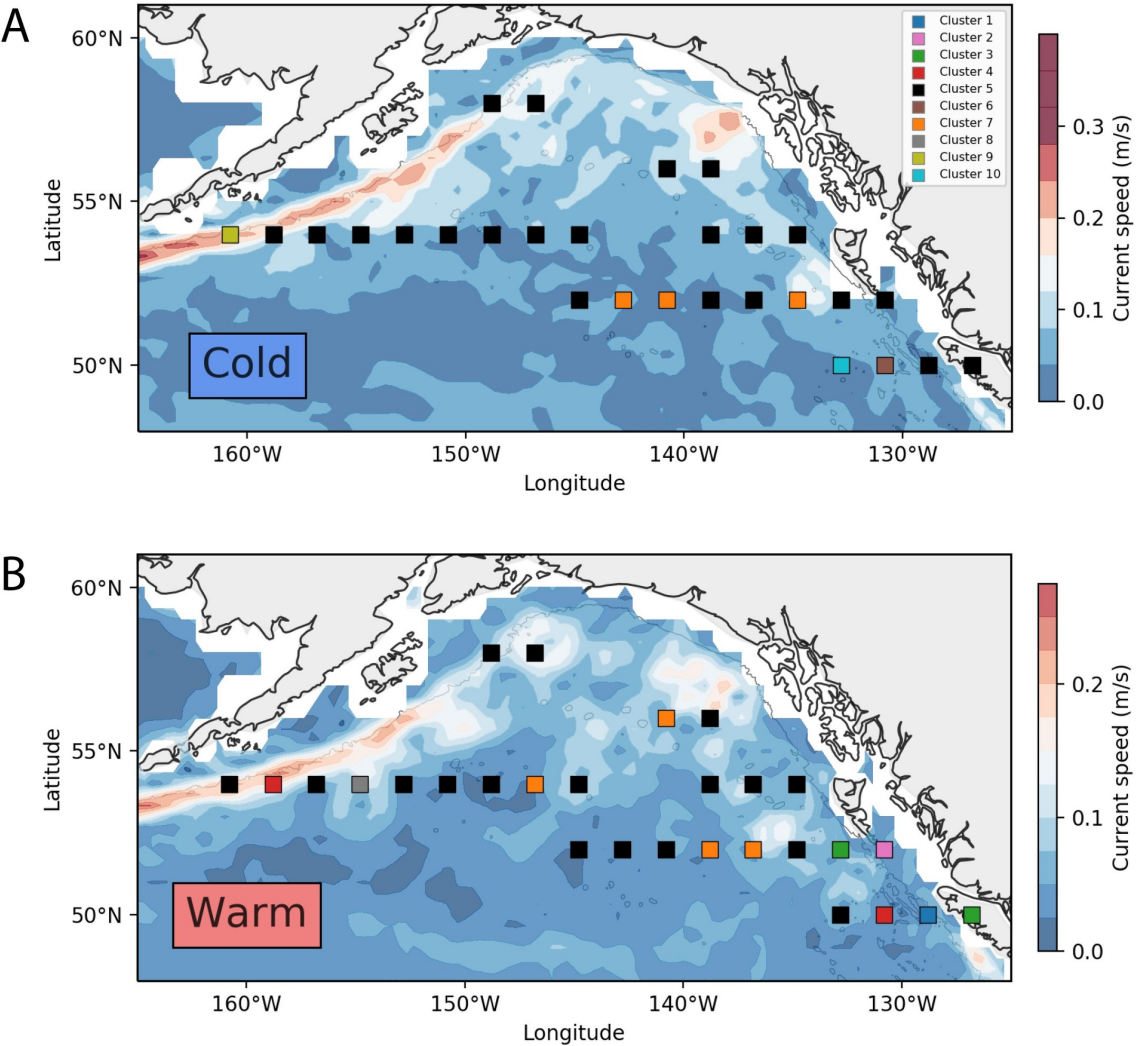
Spatial organization of clusters during years of temperature anomalies. (A) Community clusters occurring during pooled anomalously cold SST years, and their distribution relative to mean current speeds during these cold years; (B) Community clusters occurring during pooled anomalously warm SST years, and their distribution relative to mean current speeds during these warm years.

Hierarchical clustering of principle components applied on pooled years of anomalously cold and warm temperatures was conducted on ten principle components (S2 Fig) (explaining 82.4% of the overall variance) and identified ten temperature-clusters (hereafter called “Temperature-Clusters” to distinguish these assemblages from the baseline climatology clustering of the previous analysis). The spatial distribution of the temperature-clusters is depicted in Fig 9, and the association of temperature-clusters with their most significant taxa is provided in Table 4. Two of the identified temperature-clusters (Temperature-Clusters 5 and 7) occurred during both cold and warm years, and both were primarily associated with the deep GoA basin. The most common was Temperature-Cluster 5, which was characterized by a significantly lower abundance of euphausiids, chaetognaths, salps, and the copepods *C*. *pacificus* and *N*. *plumchrus*. In cold years this cluster was dominant (22 of 34 bins); other than one region adjacent to Unimak Pass in the western GoA shelf, the entire north GoA was represented by Temperature-Cluster 5 (Fig 9A). In warm years, Temperature-Cluster 5 was less ubiquitous (17 of 34 bins) and relatively more scattered in distribution. Temperature-Cluster 7 represented basin clusters with significant increases in chaetognaths, *N*. *plumchrus* and *C*. *pacificus*, and was found in the southern GoA basin during cold years (Fig 9A), and within the eastern and central GoA during warm years (Fig 9B).

**Table 4:** Taxa significantly associated with spatial clusters identified within cold or warm environmental years, 2000–2016.

Temp. Regime	Cluster	Taxa	V.test	Mean (cluster)	Mean (overall)	SD (overall)	*p-value*
WARM	1	*C*. *abdominalis*	6.87	91.5	2.88	12.9	*<0*.*001*
YEARS		*Appendicularians*	5.73	438.5	30.71	71.1	*<0*.*001*
ONLY		*Pseudocalanus spp*.	2.01	95	27.52	33.5	*0*.*04*
	2	*A*. *longiremis*	5.83	450	32.61	71.57	*<0*.*001*
	3	*Salps*	6.68	2.5	0.12	0.51	*<0*.*001*
	4	*Euphausiids*	6.44	72.12	6.26	14.16	*<0*.*001*
		*Pseudocalanus spp*.	2.61	88.75	27.52	33.50	*0*.*009*
	8	*L*. *helicina*	283.33	283.3	20.18	41.52	*<0*.*001*
		*Clione spp*.	3.33	3.33	0.55	0.68	*<0*.*001*
COLD	6	*Siphonophore*	7.39	0.75	0.02	0.1	*<0*.*001*
YEARS		*Appendicularia*	2.73	225	30.71	71.1	*0*.*006*
ONLY	9	*C*. *marshallae*	6.82	29.5	1.27	4.13	*<0*.*001*
		*E*. *bungii*	5.14	17.46	1.34	3.13	*<0*.*001*
	10	*N*. *cristatus*	5.92	134.5	11.31	20.81	*<0*.*001*
		*M*. *pacifica*	5.90	25.33	1.34	4.07	*<0*.*001*
		*Clione*	2.14	2.0	0.55	0.68	*0*.*03*
WARM	5	*Euphausiids*	-2.91	2.59	6.26	14.16	*0*.*004*
And		*C*. *pacificus*	-2.90	2.04	2.96	3.56	*0*.*006*
COLD		*Chaetognaths*	-2.58	5.38	9.17	16.51	*0*.*010*
YEARS		*Salps*	-2.55	0.01	0.12	0.51	*0*.*011*
		*N*. *plumchrus/flem*.	-2.54	41.26	54.15	56.93	*0*.*011*
	7	*Chaetognaths*	4.61	36.33	9.17	16.51	*<0*.*001*
		*N*. *plumchrus/flem*.	3.45	124.24	54.15	56.93	*<0*.*001*
		*C*. *pacificus*	3.27	7.12	2.96	3.56	*0*.*001*

^a^The means and standard deviations of each taxon within its cluster is shown, along with its positive (+) or negative (-) relationship compared to its background abundance in all other clusters.

Temperature-Clusters 1, 2, 3, 4 and 8 occurred only during warm years, and were all associated with shelf break regions along both GoA coastlines. Temperature-Clusters 1 and 2 were represented by one location each near the southeastern GoA shelf. Temperature-Cluster 1 was situated above the shelf break west of Vancouver Island, and exhibited high abundances of appendicularians and the copepods *Pseudocalanus spp*. and *C*. *abdominalis*. Temperature-Cluster 2 was north of this region, west of Haida Gwaii and the Hecate Strait, and was defined by an increase in the coastal copepod *A*. *longiremis*. Temperature-Cluster 8 occurred in the western GoA, offshore of the Alaskan Peninsula shelf break, and was characterized by increased numbers of the pteropods *Clione* spp. and *L*. *helicina*. Temperature-Cluster 3 was represented by two bins, both found in the southeastern GoA in coastal waters near Vancouver Island and Haida Gwaii, and were characterized by local increases in salp abundance. Finally, Temperature-Cluster 4 was found in deep basin waters adjacent to the shelf break on both sides of the GoA, and exhibited an increased abundance of euphausiids and *Pseudocalanus* spp.

In contrast, temperature-clusters 6, 9 and 10 occurred only during cold years. Temperature-Cluster 6 and 10 were each represented by single off-shelf locations in the southeast GoA basin. Temperature-Cluster 6, closer to the shelf, showed increases in siphonophores and appendicularians. East of Temperature-Cluster 6 and further from the shelf, Temperature-Cluster 10 was represented by *N*. *cristatus*, *M*. *pacifica* and *Clione* spp. Temperature-Cluster 9 occurred in the western GoA immediately east of Unimak Pass, synonymous with the Unimak Pass climatology cluster identified in the first analysis. This last cluster was characterized by the large deep-water copepod *E*. *bungii*, and the large shelf copepod *C*. *marshallae*.

**Fig 10:**
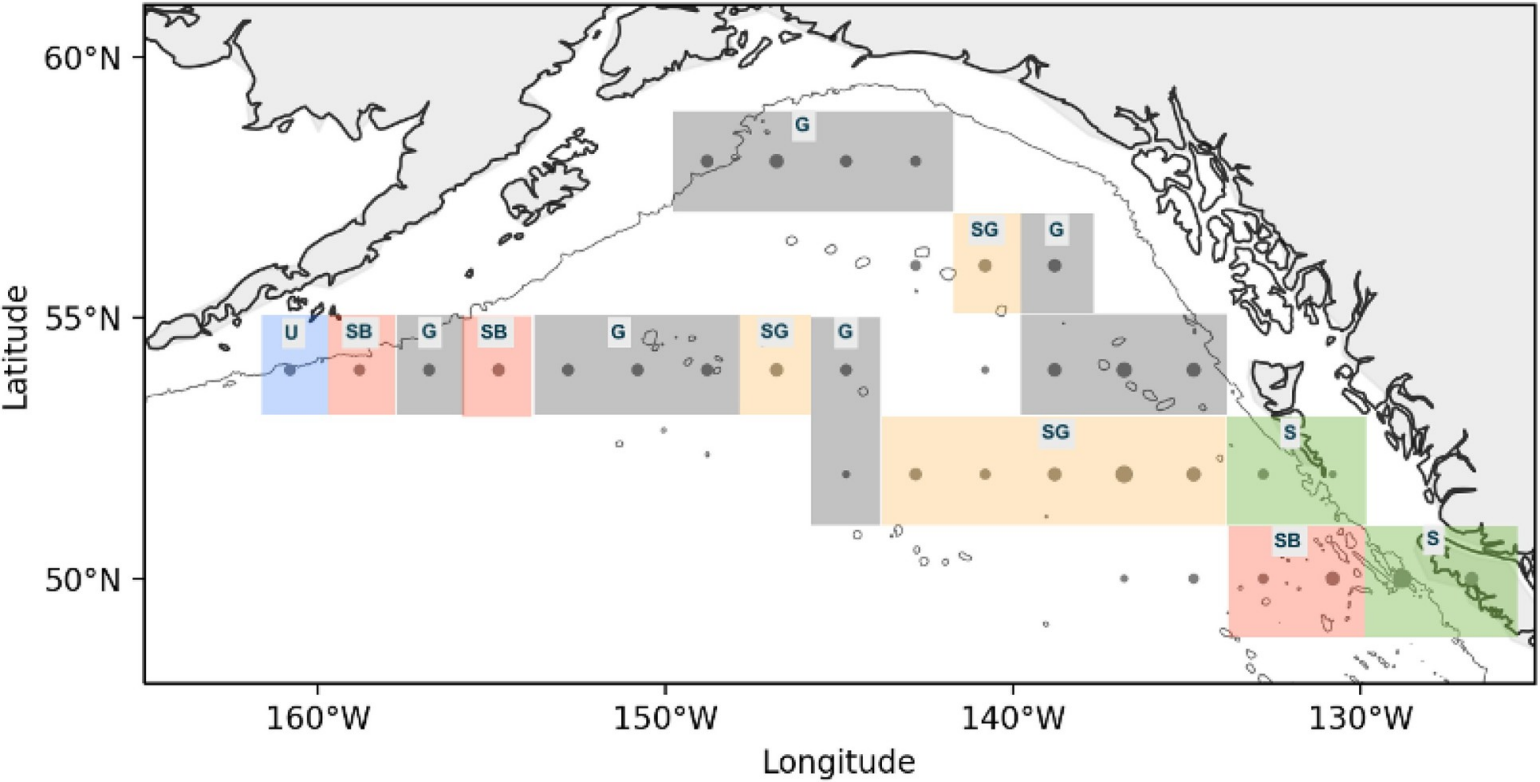
Functional similarities in community response to temperature regimes. Blocks denote regions where the zooplankton community persisted in the central Gyre (G) or shifted (SG = southern central gyre; S = shelf; SB = shelf break; U = Unimak Pass) between warm and cold years.

For clarity, the spatial patterns the Temperature-Clusters described above are summarized by their functional similarities in Fig 10. Gyre (G) blocks denote regions where euphausiids, chaetognaths, salps, and copepods N. plumchrus and C. pacificus exhibit anomalously low abundance in all years, despite temperature variation. In contrast, southern central gyre (SG) blocks are characterized by *C*. *pacificus*, *N*. *plumchrus* and chaetognaths shifting in abundance between warm and cold years. Shelf (S) blocks lie along the on-shelf eastern GoA where the coastal copepod and gelatinous zooplankton community vary strongly with temperature. Shelf break (SB) or shelf break adjacent stations also with temperature, and generally documented shifts in euphausiids and oceanic copepods. The Unimak Pass (U) block exhibited a high abundance of neritic and oceanic copepod taxa in cold years but disappeared in warm years.

## Discussion

### On-shelf and shelf break clustering patterns

Regions with strong bathymetry gradients (e.g., transitional habitats from continental shelf to deep ocean) exert strong influences on zooplankton communities. Nearshore and offshore habitats differ in hydrography, primary productivity, and benthic-sea surface coupling, and the unique oceanographic processes within each region determine local zooplankton community structure [34]. Thus, cross-shelf surveys of zooplankton communities in the north Pacific typically exhibit a gradient of neritic to oceanic species, though variability in this pattern can be strongly influenced by physical transport processes and the vertical migration and seasonal life-history traits of different zooplankton taxa [36]. In this study, we observed similar spatial dynamics that varied with bathymetry and region. For example, the most cohesive clustering separated the low abundance regions of the central gyre from all other spatial bins (Fig 3: Cluster 2), while the most divergent clusters were the shelf break off Haida Gwaii (Fig 3: Cluster 1) and along the Aleutian shelf. (Fig 3: Cluster 5). This region was characterized by the neritic copepods *A*. *longiremis* and *C*. *marshallae*. These two species, along with *C*. *abdominalis*, represent the dominant shelf copepods in the eastern GoA [37] and were found throughout the latitudinal range of our CPR sampling. *C*. *marshallae* and *A*. *longiremis* occurred broadly throughout the GoA and within all years of the study; however, these species occurred in lower numbers within deeper waters, and their elevated abundances in the southeast region are high enough for these to be classified as an indicator species. It should also be noted that the time period of this study captures different parts of the seasonal cycle for some organisms, particularly along a latitudinal gradient. Cluster 1 is in the warmest part of the study region and so likely fully encompasses the timing of peak small copepod abundance (because the CPR mesh is too large to capture many of the younger copepodite stages it is the sub-adults and adults that form the abundance peak, typically a few weeks later than the rise in juveniles would be seen). This region similarly shows a unique community assemblage during warm years (Fig 9B) but not during cold years (Fig 9A), suggesting that the sampling period may not capture the seasonal peak of small copepod abundance during cold years as effectively as during warm years.

Cluster 3 encompasses shelf and shelf break regions on both the western and eastern margins of the study domain, and is represented by increased abundances of euphausiids, salps, and the small copepods *Pseudocalanus* spp. and *C*. *abdominalis*. Among these taxa, euphausiids are often associated with high concentrations of chlorophyll-*a* and are a critical food sources for foraging fishes, seabirds, and marine mammals [38–40]. Thus, Cluster 3 suggests regions that benefit from productive shelf break processes such as coastal upwelling and fronts. The majority of the data associated with the Cluster 3 bin was collected in deep waters seaward of the shelf break, suggesting the influence of cross-shelf exchange via eddies and meandering of the Alaskan Stream [41]. Such mesoscale cross-shelf processes transport both nutrients and zooplankton, with retention times varying broadly with region and eddy strength [42, 43]. Previous investigations have reported similar effects to the results depicted in this study. Along the western GoA shelf, Alaskan Stream zooplankton surveys conducted during the productive summer season indicated local effects of cross-shelf mixing due to eddies [36]. At a broader scale, Brickley and Thomas [44] describe shelf-intensified chlorophyll-*a* around the entire basin with a significant portion of the chlorophyll-*a* variability resulting from such mesoscale features and extending up to about 300 km from the shelf break.

Among the coastal clusters, the bin adjacent to Unimak Pass in the western GoA is notable for its unique oceanographic and biological characteristics (Fig 3: Cluster 5). Cluster 5 represents one shelf break bin and one off-shelf bin in the Western GoA where the community is typified by the large cold-water copepod *E*. *bungii*, the small copepod *Oithona spp*., and the pteropod *L*. *helicina*. All of these taxa were widely distributed within the study region, and *E*. *bungii* is recognized as one of the subarctic oceanic copepods typically found off the shelf [37]. The presence of *E*. *bungii*, and the sharp contrast in community structure between the western Gulf of Alaska abyssal plain and the continental slope / shelf region near Unimak Pass, may result from the strong southerly currents and the position of Cluster 5 within the “downstream” end of the Alaska Stream which has passed the wide, productive shelf regions near Kodiak island. Batten et al. [45] also documented community partitioning in seabirds within this region, documenting an elevated diversity of seabird species from 2000–2003. The plankton data used in the Batten et al. [45] study were based on presence/absence rather than relative abundance, but again the region was distinguished by the increase of zooplankton taxonomic diversity. This region also includes parts of Cluster 2, which represents seaward shelf break habitat associated with euphausiids, salps, and the copepods *C*. *abdominalis and Pseudocalanus spp*. Therefore, the Western GoA region encompassing Clusters 2 and 5 can be seen as a “hot-spot” of diversity and abundance, supported by dynamic regional oceanography and biological productivity. Waite and Mueter report that the western shelf has the highest annual chlorophyll-*a* concentrations of the northern GoA sub-regions [46], and Sugimoto & Tadokoro found chlorophyll-*a* concentration and zooplankton biomass to be positively correlated at decadal scales, and negatively correlated at biennial time intervals due to grazing effects [47]. Increased zooplankton diversity is itself linked to intermediate zooplankton biomass levels that may optimize niche partitioning [48].

### Off-shelf clustering patterns

Compared to coastal and shelf environments, the offshore north Pacific is generally nutrient-limited and stable, with open-ocean zooplankton taxa that often exhibit strong vertical migration and mesopelagic life-history stages [42]. In comparing the eastern and western subarctic gyres, Mackas and Tsuda [49] showed the zooplankton communities to be relatively simple and similar across the NEP basin, with biomass tending to be higher along the edges of each of these gyre systems, rather than in the cores. In this study, we defined offshore regions to include the GoA deep basin and also off-shelf regions extending seaward of the continental shelf break. Two clusters (Fig 3: Clusters 2, 4) were found solely in this open ocean region, where they were differentiated not only by the composition of the oceanic zooplankton community but by opposing trends in the abundance patterns of shared taxa within these regions. Four taxa (*N*. *cristatus*, *C*. *pacificus*, *M pacifica*, chaetognaths) were significantly associated with both clusters, but whereas these taxa were abundant within the southerly distributed Cluster 4, they were depleted within the more widespread Cluster 2. Cluster 4 also exhibited significant increases in *N*. *plumchrus* and chaetognaths, signifying a significant food web contrast between the two basin clusters. *N*. *cristatus* and *N*. *plumchrus* are large and lipid-rich copepods preyed upon by a variety of predators throughout the north Pacific and Bering Sea [50–52], while the smaller *M*. *pacifica* is also known to be an important prey item for mesopelagic fishes and juvenile fish stocks [53]. For Cluster 2, however, the decreased abundance in both large *Neocalanus* copepods and euphausiids represents a significant downgrade in food web energetics for higher trophic predators in this region.

### Region and temperature effects

Regions within the Gulf of Alaska showed varying effects of temperature. For example, temperature had a limited impact in the western and northern GoA, where deep basin bins consistently identified as Temperature-Cluster 5 regardless of temperature regime. In the eastern deep basin, there was a slight expansion of Cluster 7 northwards in warm years, which conversely contained both the warm taxa copepod *C*. *pacificus* and the cold-associated *N*. *plumchrus*. These temperature-associated effects may contribute to the delineation of the two central gyre GoA baseline community clusters shown in Fig 4. Cluster 4 is spatially synonymous with Temperature-Cluster 7 (Fig 9A) in the south GoA during cold years and the northern expansion of Temperature-Cluster 7 (Fig 9B) during warm years, and both of these clusters are characterized by increased chaetognath, *N*. *plumchrus* and *C*. *pacificus* abundances. This comparison indicates that in the southeastern GoA gyre the baseline zooplankton community pattern is driven by the solidification of the Cluster 4 community (Fig 4) during cold years, yet this community shifts in warm years. In comparison, Batten and Walne [12] showed that cold-water copepod species in this region, also from CPR data, had no relationship in spatial extent with temperature, being present throughout the region in the warmest and coldest years. Warm water species, however, did show significant variability in extent, being farther north in warmer years and absent entirely in the coldest years. Under warm ocean conditions the range overlap of the two groups increases as warm water species extend northwards, causing an increase in copepod diversity. This overlap may help explain why there is less variability in community composition in the coldest years (3 unique cold year clusters versus 5 unique warm year clusters). There may also be strong variability in eddy activity and current strength/direction in the oceanic GoA during cold years, preventing an “average” composition pattern from being extracted.

Temperature effects were also seen among shelf and shelf break zooplankton assemblages. The zooplankton community of the western shelf varied strongly with temperature, with euphausiids occurring off the shelf in warm years, and *E*. *bungii and C*. *marshallae* increasing over the western GoA shelf break during cold years. The clustering patterns in this region strongly reflected the spatial shift of *E*. *bungii*, *C*. *marshallae* and *L*. *helicina* between warm and cold years; these taxa were more abundant at the Unimak Pass shelf break bin (Bin 15) in cold years (*L*. *helicina*: cold years $\bar{x}$ = 91.7, warm $\bar{x}$ = 37.5; *C*. *marshallae* cold $\bar{x}$ = 29.5, warm $\bar{x}$ = 0; *E*. *bungii* cold $\bar{x}$ = 17.5, warm $\bar{x}$ = 0), yet all three were conversely more abundant in the off-shelf Bin 18 during warm years (*L*. *helicina* cold $\bar{x}$ = 16.7, warm $\bar{x}$ = 283.3; *C*. *marshallae* cold $\bar{x}$ = 0, warm $\bar{x}$ = 0.67; *E*. *bungii* cold $\bar{x}$ = 2.0, warm $\bar{x}$ = 6.7). Thus, the shelf break region east of Unimak Pass represents a unique cold-year location, abundant in lipid-rich copepods and sharply delineated from the offshore basin. These coastal clusters overlap closely with the descending Alaskan Current, and annual variation in the speed and location of this current may influence the patterns depicted here. In contrast, the eastern GoA appeared more sensitive to temperature, with warm years causing local increases in coastal copepods and gelatinous zooplankton near the shelf, and increases in euphausiid abundance seaward of the shelf break. The ubiquitous increase in euphausiid abundance off the shelf during warm years is intriguing, as it may represent a localized trophic benefit for euphausiid predators. For example, juvenile pink salmon (*Oncorhynchus nerka*) in the GoA feed on both euphausiids and large calanoid copepods (in addition to other large zooplankton) during summer months [38, 54], particularly relying on visible zooplankton within the top surface layers (where the CPR collects data). Particularly in the bins adjacent to Unimak pass, then, the shift from nearshore *E*. *bungii* and *C*. *abdominalis* abundance in cold years towards off-shelf euphausiid abundance in warm years has strong implications for food web energetics.

The four southernmost latitudinal bins in the GoA exhibit a spatially inconsistent response to temperature, with six different clusters occurring within these bins between cold and warm years (Fig 9B). The west to east moving North Pacific Current (NPC) bifurcates into the southerly California Current and northerly Alaska Current in this region, and variability in the latitudinal position of the NPC has previously been associated with zooplankton community shifts in the GoA [55]. The variability in community shifts shown here suggest that NPC variability (speed and position) are associated with temperature change in the southern GoA, whereas this relationship does not hold in the northeast GoA.

### Mesoscale variation and climate

Previous studies have established long-term patterns in zooplankton communities linked to climate processes. For example, Hooff and Peterson [56] developed a 3-species indicator for the abundance of high nutritional value copepods in coastal Oregon, which related to the Pacific Decadal Oscillation (PDO) and salmon survival [57]. Within the eastern Pacific, Brodeur et al. found a long-term increase in overall zooplankton biomass, related to changes in winter winds in the region [58]. In this study, we did not find strong relationships between zooplankton abundance and current speeds, yet these relationships may have been masked by the stronger community-partitioning effect of temperature. The central and western GoA support our hypothesis of community persistence, whereas community variability along the Aleutian shelf and eastern Gulf, within temperature regimes, suggest the influence of mesoscale physical processes such as eddies and gyres. In the north Pacific, coastal anti-cyclonic eddies provide off-shelf transport of heat, nutrients and plankton, therefore variability in these physical processes can strongly affect the distribution of surface plankton. Eddies can also affect the vertical distribution of zooplankton by structuring local primary production and altering the behavior of diel migrating taxa [59]. In the north Pacific, the results of this study support meso-scale associations between temperature, currents and zooplankton communities. For example, the station nearest Unimak Pass is identified as a productive cold-water cluster with an elevated abundance of the oceanic *E*. *bungii* and the shelf resident *C*. *marshallae*, indicating increased cross-shelf mixing, yet this cluster dissipates entirely during warm years when the current speed of the Alaskan Current slows. Cross-shelf exchange along the western GoA has been previously linked to increased abundance in *E*. *bungii* and *C*. *marshallae* [36, 60], though *C*. *marshallae* have also been shown to be more abundant in warm years [36]. Biologically, the dissipation of the *E*. *bungii* / *C*. *marshallae* cluster in warm years could partially reflect earlier diapause in *E*. *bungii* (August and later) [61] and an earlier maturation and diapause of C1-C5 *C*. *marshallae* (generally July-Sept. in Bering Sea) [62]- this would reduce the abundance of C4-C6 stage copepods caught in the survey mesh. The pattern could be associated with a weaker or re-positioned descending Alaskan Stream current that spins off fewer and weaker surface eddies during warm years, reducing cross-shelf exchange. While the mechanisms between SST and western GoA eddy dynamics remain under active investigation, the increased nutrient availability facilitated by cross-shelf exchange is likely to be the strongest predictor of the coastal patterns we observe here [63, 64].

The Alaska Current is the primary oceanographic feature in our study area, and weak circulation of the current in the Subarctic gyre has been previously associated with warmer and more stratified conditions [65], reducing primary productivity and fisheries yields [66]. While variability in the strength and timing of North Pacific hydrography ultimately derives from atmospheric pressure systems in the North Pacific, the flow of the Alaska Current has now been shown to also vary with the latitudinal position of the North Pacific Current where the Alaskan Current diverges northward, and the Pacific Decadal Oscillation [67]. Intriguingly, in our study this region off coastal Vancouver Island was also the most variable region in terms of zooplankton community persistence, even within temperature regimes. Latitudinal variation in the NPC may affect current flow and thus zooplankton communities throughout the coastal domains of the GOA, with downstream effects on salmon stocks and trophic energy flow [68].

## Conclusions

In summarizing 16 years of data temporally and across 2 degree cells, we may be smoothing anomalously high abundance values that occur within specific years or locations, and obscuring some taxa signatures of interest. However, the overall pattern of the results shown here are consistent with previous studies in the region using short-term CPR data, and uphold our hypothesis that there is large-scale persistence in the spatial distributions of zooplankton communities, particularly in the deep central and western GoA. It is notable that this effect persists even though the study period encompassed a wide range of ocean climate conditions, including some of the coldest SST years in recent decades as well as the strongest global marine heat wave on record. Establishing a community climatology throughout the north Pacific, as this study has done, reveals regions of stable community persistence versus regions of community structure variability that are associated with high frequency variability in current and eddy structure. Given the warming trends in the north Pacific, regions that showed high variability between temperature regimes are likely to shift towards the warm-year assemblage patterns depicted here. For example, warm-year taxa are likely to increase in the eastern GoA, whereas cold-water assemblages located along the Aleutian shelf might decrease in occurrence. Given the abundance of large, lipid-rich copepods in the cold-water Aleutian shelf region, climate shifts are likely to have enduring impacts on the food web in this area. The spatial variation that exists within warm and cold years, particularly along the highly variable south GoA, suggests that other factors, such as mesoscale hydrography, play a larger role than just temperature alone.

## Supporting information

S1 Fig PC selection model output for overall zooplankton community climatology. Model output illustrating the optimal number of components to keep for the baseline community climatology cluster analysis. This graphic was one of 2 methods used to determine the appropriate number, with both methods suggesting the retention of 5 components.
(DOCX)

S2 Fig PC selection model output for temperature-mediated zooplankton community climatology. Model output illustrating the optimal number of components to keep for the baseline community climatology cluster analysis. This graphic was one of 2 methods used to determine the appropriate number, with both methods suggesting the retention of either 10 or 15 components.
(DOCX)

S1 Table Output from permutational multivariate analysis of variance using distance matrices (ADONIS). Table results depict overall community difference between cold and warm years using temperature and depth.
(DOCX)
