# Supplementary material for: Spatio-temporal persistence of zooplankton communities in the Gulf of Alaska
Source: PLoS One. 2021 Jan 22;16(1):e0244960. doi: 10.1371/journal.pone.0244960 (PMC7822315; doi:10.1371/journal.pone.0244960)

**S2 Fig. PC selection model output for temperature-mediated zooplankton community climatology.** Model output illustrating the optimal number of components to keep for the baseline community climatology cluster analysis. This graphic was one of 2 methods used to determine the appropriate number, with both methods suggesting the retention of either 10 or 15 components.


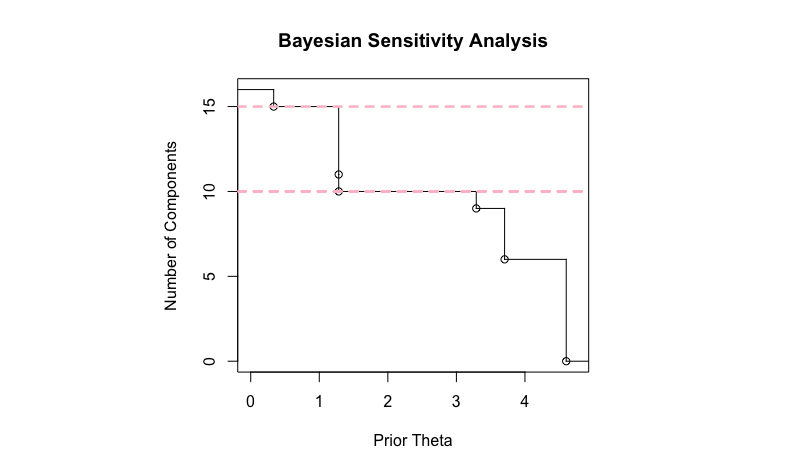

Supplement: S2 Fig — Model output illustrating the optimal number of components to keep for the baseline community climatology cluster analysis. This graphic was one of 2 methods used to determine the appropriate number, with both methods suggesting the retention of either 10 or 15 components. (DOCX) [file pone.0244960.s002.docx]
