# Supplementary material for: Spatio-temporal persistence of zooplankton communities in the Gulf of Alaska
Source: PLoS One. 2021 Jan 22;16(1):e0244960. doi: 10.1371/journal.pone.0244960 (PMC7822315; doi:10.1371/journal.pone.0244960)
Supplement: S1 Table — Table results depict overall community difference between cold and warm years using temperature and depth. (DOCX) [file pone.0244960.s003.docx]

**S1 Table: Output from permutational multivariate analysis of variance using distance matrices (ADONIS), testing for overall community difference between cold and warm years using Temperature and Depth.**

|  | Df | SS | MS | F.model | R2 | *p* |
| --- | --- | --- | --- | --- | --- | --- |
| Temp | 1 | 0.24 | 0.24 | 1.12 | 0.012 | *0.35* |
| Depth | 1 | 0.78 | 0.78 | 3.63 | 0.06 | *0.001* |
| Temp:Depth | 1 | 0.63 | 0.64 | 2.96 | 0.05 | *0.004* |
| Residuals | 52 | 11.13 | 0.21 |  | 0.87 |  |
| Total | 55 | 12.8 |  |  | 1 |  |
